# Exploring and Mapping Expert Views on the Mechanisms Contributing to Patients’ Demand for Low-Value Care: A Complex Systems Approach

**DOI:** 10.34172/ijhpm.9099

**Published:** 2026-06-01

**Authors:** Gillroy R. L. Fraser, G. Ardine de Wit, Job van Exel, Peter M. A. Sloot, Raymond W. J. G. Ostelo, Frenk van Harreveld, Mattijs S. Lambooij

**Affiliations:** ^1^Department of Health Economics and Health Services Research, National Institute for Public Health and the Environment (RIVM), Bilthoven, The Netherlands.; ^2^Department of Health Sciences, Faculty of Science, Vrije Universiteit Amsterdam, Amsterdam Public Health Research Institute, Amsterdam, The Netherlands.; ^3^Erasmus School of Health Policy & Management, Erasmus University Rotterdam, Rotterdam, The Netherlands.; ^4^Erasmus Centre for Health Economics Rotterdam (EsCHER), Rotterdam, The Netherlands.; ^5^Institute for Advanced Study, University of Amsterdam, Amsterdam, The Netherlands.; ^6^Independent Complex Systems Researcher, Noordwijk, The Netherlands.; ^7^Department of Epidemiology and Data Science, Amsterdam University Medical Centre, Vrije Universiteit, Amsterdam, The Netherlands.; ^8^Department of Psychology, Faculty of Social and Behavioral Sciences, University of Amsterdam, Amsterdam, The Netherlands.; ^9^Center for Prevention, Lifestyle and Health, Department Behaviour & Health, National Institute for Public Health and the Environment (RIVM), Bilthoven, The Netherlands.

**Keywords:** Low-Value Care, Complex Systems Approach, Causal Loop Diagram, Demand for Care, Patient Preferences for Overuse

## Abstract

**Background:**

Approximately 20% of the total healthcare expenditure in high-income countries is spent on low-value care, ie, care that is unnecessary, potentially harmful, or provides marginal health benefits to patients. Demand for low-value care is considered a multifactorial, complex problem, as a multitude of factors have been associated with low-value care use. However, there is limited knowledge on how these factors interrelate and lead to demand for low-value care. Therefore, the aim of this study was to explore and map the factors and their relations contributing to patients’ demand for low-value care using a complex systems approach.

**Methods:**

Two group model building (GMB) sessions on the topic of low-value care were organised with experts from the Netherlands. Each session’s transcript was thematically analysed, resulting in one causal loop diagram (CLD) per session. These CLDs included determinants, relations between these determinants and feedback loops. Finally, the CLDs were synthesised into one CLD combining the insights from both sessions.

**Results:**

The final CLD consisted of 42 factors influencing demand for low-value care. It includes biomedical factors, cognitive biases, socio-cultural factors, economic factors, emotions, knowledge-related factors, factors related to the interaction with the provider, and preferences and expectations. By mapping the relations between these factors, we identified 59 connections and nine reinforcing feedback loops potentially influencing demand for low-value care.

**Conclusion:**

The CLD provides insight into factors, mechanisms and feedback loops influencing patients’ demand for low-value care. It highlights perceived insecurity as a central driver that influences multiple other factors and eventually affects patients’ demand for low-value care. These central factors influencing multiple other factors may be potential leverage points for policies aiming to reduce demand for low-value care. Further research is required to clarify the relative importance of the identified factors, relationships, and feedback loops to determine effective leverage points.

## Introduction

**Key Messages**

**Implications for policy makers**

- Patients’ demand for low-value care is a multifactorial and multilevel problem, influenced by clinicians, system-level factors, and patients.
- This study provides policy-makers with insights into the factors and relations driving patients’ demand for low-value care.
- These insights can help policy-makers to develop and implement effective interventions.
- Potential interventions could target the underlying beliefs and central drivers (eg, perceived insecurity) to influence multiple factors and feedback loops, for example through clinical guidelines and public education campaigns aimed at reducing uncertainty-driven demand.
- Policy-makers could also target feedback loops (eg, loops R1-R9) or factors within these loops to disrupt or reduce their effects on other factors and diminish demand for low-value care.

**Implications for the public**

Some interventions are unnecessary, potentially harmful, or offer little to no health benefits for patients. These interventions are referred to as low-value care. Despite being discouraged, patients often continue to demand it. This study may help patients understand the factors and relations driving low-value care demand. By recognizing these factors and relations, patients may become more aware and cautious of demanding unnecessary or harmful care. This awareness could improve health outcomes by encouraging patients to avoid low-value care and follow evidence-based recommendations. Over time, it may also foster sustainable new habits and beliefs that reduce demand for low-value care among patients.

Low-value care, defined as medical services that are unnecessary, potentially harmful, or provide no or marginal health benefits to patients,^1^ is a global problem.^1-5^ Examples include routine imaging for nonspecific low back pain, prescription of unnecessary antibiotics, and PSA-testing for patients older than 69 years.^6-8^ Estimates suggest that in Organisation for Economic Co-operation and Development countries like the United States and the Netherlands, at least 20% of healthcare expenditure concerns low-value care.^1^ To reduce low-value care, researchers have studied factors that contribute to the use of low-value care.^1,3,8-10^ Most of these studies focused on factors that explain why medical practitioners provide low-value care services. These factors are referred to as supply-side factors.^11,12^ Examples of supply-side factors include financial incentives, fear of medicolegal consequences when failing to detect or diagnose a condition, preference for doing something rather than doing nothing, and medical practitioners’ anticipation of patient preferences, which can lead to the provision of low-value care.^3,12-15^

 Besides physicians, patients have also contributed to low-value care use. While it may seem counterintuitive for patients to demand services that may be harmful or provide little to no value to them, the literature provides different rationales for this.^16-22^ These include psychological factors, such as cognitive biases, emotions, preferences and expectations, but also knowledge-related factors, biomedical and care-related factors, socio-cultural factors, economic factors, and factors related to the interaction with providers.^22^ Often these factors are studied within a disease-specific context and as separate, independent factors contributing to low-value care use, making it challenging to understand how these factors influence patients’ demand for low-value care.^3,23,24^ However, it seems likely that these factors are not independent but interact with one another.^3,23,24^ Accordingly, several researchers examined some relations between factors and their potential synergistic effects on low-value care use, and have referred to low-value care use as a multifactorial or “complex problem.”^2,3,10,23,25,26^ Nevertheless, these examined relations have not yet been mapped or thoroughly explored, which also appears to be the case specifically for patients’ demand for low-value care.^3,22,25^ Understanding the relations between various factors and their complex interplay contributing to low-value care demand seems crucial and should be a priority to develop effective interventions to reduce this demand. Therefore, the aim of this study is to explore and map the factors driving patients’ demand for low-value care and the relations between them, by using a complex systems approach.

## Methods - Group Model Building and Causal Loop Diagram

 To better understand the demand-side factors driving low-value care and their relations, we organised two online group model building (GMB) sessions with experts from the Netherlands. GMB is a method used to understand complex problems by eliciting participants’ knowledge, including their thoughts, feelings, and experiences about how things work in a specific context. This process is also referred to as capturing their “mental models.”^27^ Discussing these mental models allows participants to engage in group learning and develop a shared understanding of the complex system under discussion.^28^ Based on each GMB session, we constructed a causal loop diagram (CLD). A CLD is a conceptual model or visualisation of the combined mental models of the participants.^29-31^ The CLD captures the combined knowledge of experts on factors driving patients’ demand for low-value care and the (causal) relations between these factors.

###  Scripts

 To conduct the GMB sessions and construct the CLDs we used available scripts from Scriptapedia (https://en.wikibooks.org/wiki/Scriptapedia).^32-36^ These scripts provide structured activities to guide group processes and introduce participants to complex systems, causal mapping, and feedback loops.

###  Reducing Cognitive Load on Participants

 Dividing participants’ attention between the discussion (ie, listening, reflecting, and responding to others’ input) and live CLD visualisation may increase cognitive load and hinder their ability to process information, contribute effectively, and achieve shared understanding.^37^ To reduce split-attention effects and minimize participants’ cognitive stress or overload, we used a stepwise approach to construct the CLD and avoided live CLD visualisation during the GMB session.^37,38^ After the in-depth discussions in each GMB session, the authors drafted a CLD based on the transcript of each discussion (step 1). In the second step, we asked participants to validate the draft CLDs. In the third step, we combined the draft CLDs of each GMB session and asked the participants to validate the synthesized CLD.

###  Participants

 Prior to the study, we conducted a scoping review of the literature on factors contributing to patients’ demand for low-value care (See Supplementary file 1).^22^ Based on the identified factors and actors in this review, we constructed a list of potential occupations relevant for the GMB sessions. We used this list and invited experts with different backgrounds in the Dutch healthcare sector to participate in the GMB sessions (See Table).^32,35^ Participants for the first session were recruited through purposive sampling. Experts were selected because of their familiarity with low-value care, face to face experience with patients demanding low-value care, background in healthcare insurance, publications on risk communication, public health, medical decision-making, and low-value care. At the end of the first session, we used snowball sampling to gain insight into potential participants for the second session.^36^ Participants were asked to recommend specific individuals or backgrounds of experts that were not yet included in the study. We stopped organising GMB sessions when no new participants or backgrounds were recommended.

**Table:** Expert Domain Table

**GMB session 1**	**Fields of Expertise**
Participant A	Physician, researcher appropriate care and low-value care
Participant B	Physician, healthcare insurance advisor, medical management consultant
Participant C	Researcher/social psychologist in the field of medical decision-making and complex systems sciences
Participant D	Cognitive psychologist and health behaviour researcher
**GMB Session 2**	**Fields of expertise**
Participant E	Low-value care researcher, health economics researcher
Participant F	Researcher public health, chronic diseases, and health behaviour
Participant G	Researcher public health risk communication, and medical decision-making

Abbreviation: GMB, group model building.

 We considered inviting patients and patient representatives as well but concluded that this would be more relevant in a disease-specific case-study. Patients can obviously contribute valuable information about what influences their demand for low-value care in the context of a specific disease. At this stage, however, involving a sufficiently large and varied group of patients to obtain information on factors and relations that are relevant across specific contexts was considered not to be feasible.

###  Information Provided to Participants

 Before participating in the session, participants received information about the objective and setting of this study.^32-34^ The organiser (GRLF) and moderator (PMAS) contacted each participant individually to provide details about the study. To assure mutual understanding of the topic between participants, we explained the definition of low-value care in the current study (ie, medical services that are either potentially harmful, unnecessary, or provide little to no health benefits to patients).^1^ We stated that patients may not explicitly ask for low-value care from their provider but may demand more care than needed or unintentionally demand potentially harmful care.

 Secondly, we specified that we were interested in identifying factors that contribute to patients’ demand for low-value care, the relations between them, and feedback loops. We described feedback loops as a circularly linked set of factors that are presumed to be each other’s causes and effects. These loops indicate how a change in one factor induces changes in other factors, subsequently affecting and altering the original factor itself. Identifying feedback loops is important because they can shift the entire system away from its initial state (ie, reinforcing loops) or back towards its initial condition (ie, balancing loops).^39,40^ Participants were instructed not to focus on how long it takes for one factor to affect another, as our focus was on identifying factors and relationships.

 Furthermore, we encouraged participants to engage in exchanges and debates about their mental models on the topic during the session, and to seek consensus between them.^27^ Participants were informed of the role of the moderator to guide the sessions,^35^ how the CLD would be created, and the post-session validation of the CLD.^35^

 Finally, participants were informed about the anonymisation and removal of their personal information from this study. We included only participants’ fields of expertise and the information needed to identify factors and relations, answered the remaining questions, and obtained participants’ informed consent.

###  Topic List 

 Prior to the study, we conducted a scoping review of the literature on factors contributing to patients’ demand for low-value care.^22^ Based on the factors identified in this review, we created a topic list for the GMB sessions. The topic list was not shared to limit its influence on the participants and to allow them to make as many free associations as possible based on their own knowledge and experiences.

 The moderator used the topic list for the following four purposes. Firstly, to prepare for the sessions, as potential material for stimulating discussion or for posing follow-up questions that could encourage participants to delve deeper into their reasoning. Secondly, at the start of each session, one randomly selected factor was used as an example to familiarise the experts with the GMB process. Thirdly, the topic list was used at the end of each session to introduce one or two factors that were identified in the literature (See Supplementary file 1). This was done to assess the relevance of these factors relative to those discussed spontaneously and to identify whether introducing these factors would prompt additional discussions. Lastly, when experts provided examples that could be connected to multiple demand factors on the topic list, the moderator mentioned these factors. Participants where then asked to choose the most fitting factor for each example, which improved clarity during the sessions. The same was done to capture the names of most feedback loops. This approach integrated participant input with existing evidence from the literature, providing the factors in the CLD with a literature-informed foundation (ie, incorporating terminology from the literature corresponding to participant input).

###  Knowledge Elicitation

 During the GMB sessions we applied various knowledge elicitation techniques to draw out ideas and capture experts’ mental models regarding low-value care demand. We started by implementing the “round robin brainstorm technique” and dedicated time for each participant to answer the question:* “Can you name factors associated with patients’ demand for low-value care?”*^41^ This question was asked to identify the factors that participants were aware of and to provide an initial set of factors for the discussion. Next, we asked two follow-up questions to each participant: “*Can you explain how these factors interact with each other and eventually result in patients’ demand for low-value care?”* and *“Can you identify any feedback loops?” *Each participant answered these questions individually before engaging in a group discussion.

 Initial agreement between participants, without discussing the factors or their relations, may indicate a lack of an in-depth discussion. For these instances, techniques such as “playing the devil’s advocate” were used to promote discussion and highlight potentially conflicting viewpoints.^27,42^ In some cases, factors and relations brought up by an expert were overlooked and not discussed by the group. In these cases, we used “the parking lot technique.”^32^ Overlooked factors or relations were recorded, temporarily “parked” by the moderator, and at a later point repeated to the group for discussion. At the end of each session, the moderator used the topic list to highlight randomly selected factors that were not mentioned by the participants. This was done to confirm that we had reached saturation in the discussion, and no new factors or relations would come up. To verify this, the moderator asked each participant one last time if they would like to add anything to the discussion.

###  Thematic Analysis

 We recorded and transcribed the GMB sessions verbatim. The transcripts of each session were thematically analysed by one of the authors (GRLF) to extract a CLD.^43,44^ Using open coding,^45^ text fragments containing factors that contribute to patients’ demand for low-value care were translated into English, highlighted, analysed and summarised in one or a few words. Factors similar to those in the review of Fraser et al^22^ received the same names. New factors were defined based on the content of the text fragments in which they appeared. Next, we focused on identifying and understanding the relations between factors. This was done by examining how participants connected factors to each other using certain signal expressions, such as “results in,” “leads to,” “is caused by,” “a consequence of,” and “has an effect on.”^44^

 We created a “words-and-arrow diagram”^43^ to visualise the identified relations by linking factors to each other and by specifying the polarity (ie, positive or negative relation) for each relationship as indicated by the experts during the sessions (See Supplementary file 2). The “words-and-arrow diagrams” present multiple isolated overviews of identified relations during various moments in the discussion. After completing this process for each session, we (GRLF, GAdW, and MSL) reviewed the data to validate the interpretation.

###  Constructing the Causal Loop Diagrams

 The structure of the “words-and-arrow diagrams” was used as a baseline to create a CLD for each GMB session. In this process, we adhered as much as possible to connections made in the “words-and-arrow diagram” but deviated from it in a few cases. For example, when experts initially linked factors directly to patients’ demand for low-value care, but later in the session in an indirect, more elaborate manner. In these cases, we recorded all involved factors and relations in the “words-and-arrow diagrams” and prioritised the indirect relationships when constructing the CLDs. Direct relations mostly reflected the experts’ initial thinking, whereas indirect relations often emerged after deliberation. We considered these indirect relations to be of higher quality (ie, more detailed, and richer in information) and prioritised them in the CLDs. Direct relations where still included when no additional information was provided.

 Experts stated that “demand for low-value care” should be a factor in the CLD, as demand, if developed into a customary practice, can influence more patients to demand low-value care. Including “demand for low-value care” as a factor in the CLD may be seen as paradoxical, as it is an emerging variable created by the interaction between numerous factors. Placing an emerging variable in a mapping of its underlying structure should be avoided.^46^ However, factors in complex systems can interact with emerging variables. Therefore, we decided to acknowledge the view of the respondents and included “demand for low-value care” in the CLD. Lastly, we asked participants to validate the CLD that was constructed based on their session. The participants were contacted for a one-on-one session. In this session, the modeller explained the CLD, and participants could provide feedback. The feedback was evaluated and processed.

###  Synthesising the Causal Loop Diagrams

 We merged the CLDs from the GMB sessions into one final CLD to obtain a comprehensive overview of the identified factors and their relationships. Common factors, connections, and feedback loops were used to build its basic structure. Subsequently, unique factors and relations from the individual CLDs were added into the final CLD. We verified whether relations that differed across the individual CLDs were represented in the final CLD. Remaining inconsistencies between the structure of the final CLD and the separate CLDs were corrected by adding the missing connections or by following the connections of the CLD that offered a more in-depth explanation. This process was discussed with all authors. Next we shared the final version of the CLD with the participants to receive their feedback.

###  Literature-Based Validation

 Some participants provided literature references supporting factors and relationships mentioned during the session(s) and illustrated in the CLD. To substantiate other factors and relationships, we examined the literature on patients’ demand for low-value care and included applicable references in the results. However, the majority of existing studies did not explore causal relationships and often focused on identifying associations between factors. Hence, these studies generally did not examine the polarities and directions of relations between factors. Therefore, our inclusion criteria encompassed references that explicitly identified factors and their relationships in the CLD. We also included references that implied such relations through written descriptions or conceptual discussions, even when the polarity of the relationships was unspecified. All factors and relations were retained in the CLD, to maintain the causal structure as identified by the participants. Factors and connections were not quantitively weighted to distinguish their importance, and none were excluded based on weak or conflicting evidence from the literature.

## Results

###  Participants

 A total of seven experts participated in two GMB sessions (See Table), four (experts A-D) in the first session and three (experts E-G) in the second. Table provides information on the professional background and expertise of experts involved in the GMB sessions. All participants were invited to provide feedback on the CLDs developed during their session, but only three participants (A, C, and E) volunteered. These participants also provided validation feedback for the final CLD, while the remaining participants were unavailable.

###  The Final Causal Loop Diagram

 The final CLD consisted of 42 factors (See Figure 1) that were categorised in eight overarching themes, namely cognitive biases, emotions, preferences and expectations, knowledge-related factors, socio-cultural factors, system factors, biomedical and care-related factors, economic factors, and factors related to the interaction with the healthcare provider (See Supplementary file 3, for the definition of each factor). Furthermore, we identified 59 causal connections between these factors (See Figure). These connections were either positive (51 out of 59 connections; presented as black arrows) or negative (8 out of 59 connections; presented as red arrows). Factors that were positively connected to each other moved in the same direction, implying that when one factor increases or decreases, the other factor increases or decreases as well. In contrast to this, factors that were negatively connected moved in opposite directions. A decrease of one factor results in an increase of the other linked factor and vice versa. The final CLD includes nine reinforcing loops: (R1) “Uncertainty avoidance,” (R2) “Regret aversion,” (R3) “Shopping around,” (R4) “Interdependent anticipation,” (R5) “Social-cognitive influences,” (R6) “Practice-induced demand,” (R7) “Care entitlement,” (R8) “Social and media influence,” and (R9) “Patient-provider relationships.” All feedback loops were positive in the final CLD and are represented by the blue texts in Figure. No negative feedback loops were identified.

**Figure:**
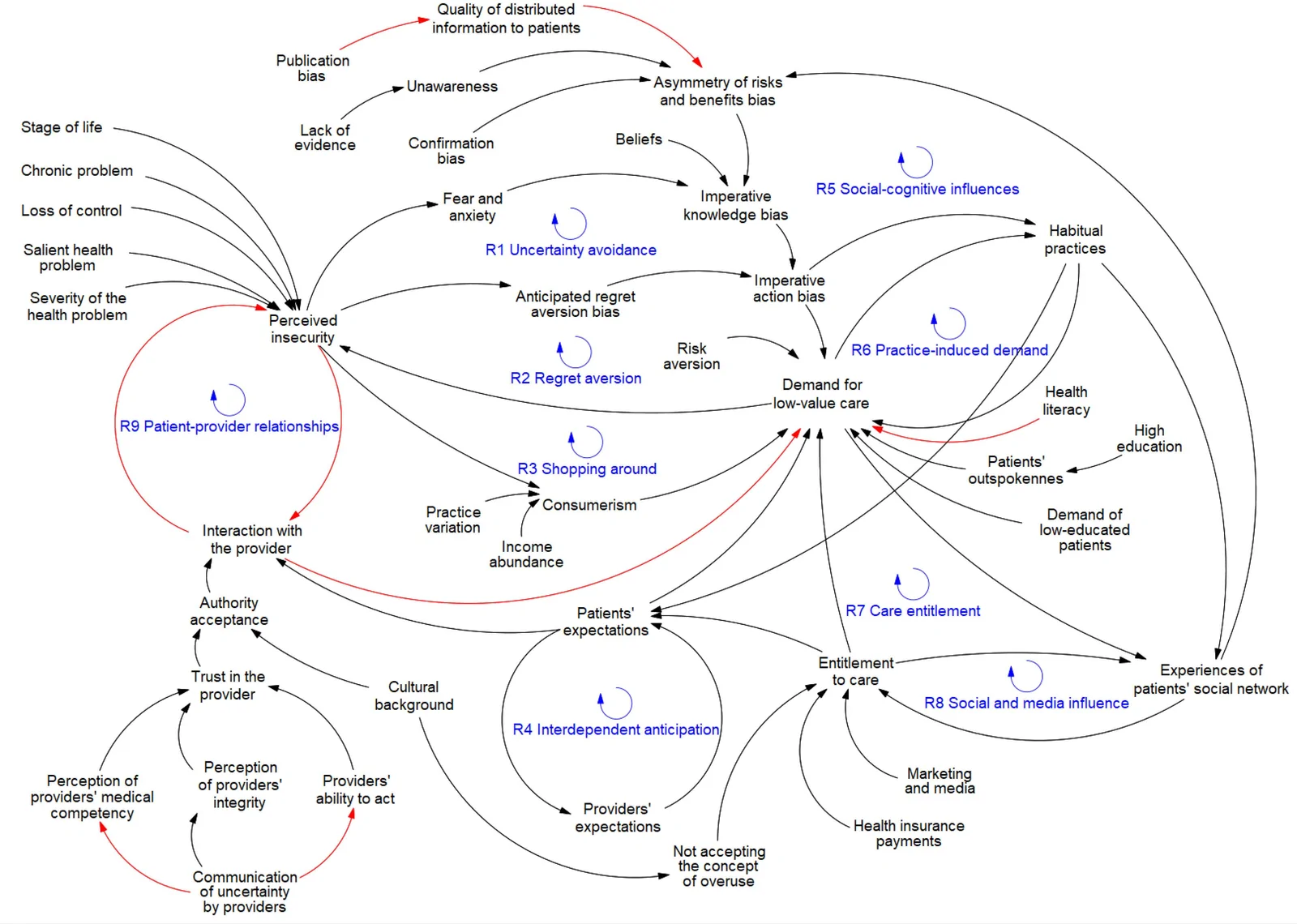
Final Causal Loop Diagram.

 The authors discuss each loop and factor, using examples and situations mentioned by the participants during the GMB sessions. Please note that these examples may be context specific and, therefore, not generally applicable. Figure contains a complete overview of the CLD.

 Regarding loop R1 “Uncertainty avoidance,” participants concluded that patients could demand low-value care to reduce or avoid health-related uncertainties.^47-49^ This loop is interpreted from left to right and starts with five factors that were perceived to be potential causes for patients to feel insecure and uncertain about their health status. Participants stated that when patients become more insecure, they also become more fearful and anxious. As a result of this, patients could believe that knowing more about their health status is better than knowing less (imperative knowledge bias). Participants mentioned that some patients may have this bias without specifically understanding its cause. For them, the loop would start directly from this point (imperative knowledge bias). Next, the desire to know more about their health status can also drive patients to think that it is better to do something rather than nothing (imperative action bias), which can then result in demand for low-value care.

 Participants stated that in most cases patients were initially denied access to the low-value care that they demanded from their healthcare provider. As a result of not receiving this care, patients could experience an increased level of insecurity, because they did not gain any comforting information about their health status. On the other hand, patients may receive the care that they demanded. However, when such care does not improve their health status, as is expected of low-value care, then this may also increase their perceived insecurity.^50^ Either way, the loop starts again and continues in a cyclical manner, constantly increasing the level of perceived insecurity, fear and anxiety, imperative knowledge bias, imperative action bias, and demand for low-value care. Although the factors in the loop remain the same, their intensity will change over iterations of this loop. Therefore, the effects of loop R1 on the state of the entire CLD (See Figure) may increase with each iteration of this loop. This finding is applicable for all reinforcing loops (R1 to R9).

 In loop R2 “Regret aversion,” anticipated regret can incentivise patients to demand low-value care.^10,51,52^ The starting conditions of this loop are similar to the ones described in loop R1. When patients’ perceived insecurity increases, the tendency to avoid regret in the future becomes stronger (anticipation regret aversion bias), and patients are more likely to think that taking action is better than inaction (imperative action bias).^53^ As a result of this, participants expected patients to demand more low-value care. As in loop R1, participants believed that this demand for low-value care might, in turn, reinforce patients’ insecurity and, therefore, the connection between “demand for low-value care” and “perceived insecurity” was expected to work in the same reinforcing manner.

 Loop R3 “Shopping around” captures the idea that patients may perceive healthcare as a consumption good. If so, patients will visit multiple healthcare providers until they receive the care that they desire.^47,54,55^ Three factors were identified and linked to this behaviour (consumerism).

 First, people experiencing insecurities caused by symptoms or health-related risks were assumed to shop around more for low-value care.^47,56,57^ This may concern patients who are actually ill, but also people who have not been diagnosed with any illness or do not experience any symptoms. For example, people who have reached a certain age (stage of life) are commonly associated with increased health risks and can experience insecurity about their health.^58,59^

 Secondly, participants expected that practice variation could result in more consumerism and, subsequently, increased demand for low-value care. Healthcare providers can recommend differently despite similar circumstances or patient traits.^60,61^ Greater practice variation provides patients seeking care with more care options.

 Thirdly, participants argued that income abundance could increase demand for low-value care indirectly. People with more financial means were expected to be willing to spend more money on desired care. As a result, they may display more consumerism and demand more low-value care.^62^ As in the previous two loops, demand for low-value care increases the level of perceived insecurity, making R3 a reinforcing loop. For example, total body scans may result in false positive findings, reinforcing experienced insecurity and, subsequently, consumerism.

 Loop R4 “Interdependent anticipation” can be interpreted from either the patient’s or the healthcare provider’s perspective. On the one hand, patients can feel entitled to demand low-value care and a stronger sense of entitlement can increase their expectations of receiving care.^55,63^ Patients expressing their expectations to receive care can, in turn, influence the expectations of their healthcare provider about patients’ demand in future instances. On the other hand, it was noted that patients’ expectations may be influenced by the expectations of their healthcare provider.^4,59,64,65^ From this viewpoint, patients tend to accept care that their provider recommends. Participants argued that this may also apply to healthcare providers, when they base their recommendations on what they expect patients want to receive. For example, physicians may anticipate that their patients expect to receive unnecessary care, even when this is not needed or recommended.

 Participants concluded that intensification of these mutual expectations will result in more demand for low-value care. The reinforcing effect of patient-provider expectations also has an impact on the whole CLD, and influences patients’ demand for low-value care in a direct manner and other factors indirectly through connected mechanisms.^10,66-68^

 Loop R5 “Social-cognitive influences” describes the interplay between patients’ cognitive processes and their social environment. Interpreting this loop from the effect of “demand for low-value care” on the “experiences of patients’ social network,” increased demand for low-value care on the societal level may transfer to individuals within social networks of patients.^51,69-72^ The experts expected experiences shared by patients’ social networks to influence risk perceptions of patients, leading patients to overestimate benefits and underestimate risks of preferred care (asymmetry of risks and benefits bias).^16,73^

 In addition to the experiences from social networks, three other factors were described to affect patients’ asymmetry of risks and benefits bias. These factors were “confirmation bias,” “patients’ unawareness,” and the “quality of distributed information to patients.” First, patients influenced by the confirmation bias seek and interpret medical information corresponding to their viewpoints and neglect or disagree with counterintuitive information.^74^ Second, patients lacking evidence-based information are unaware of the potential health risks of unnecessary care and could underestimate risks associated with low-value care interventions.^3^ Third, overemphasising positive results about the effects of care interventions in research articles or by the media (publication bias), could cause the quality of distributed information to diminish.^3,75,76^ Patients using these low-quality sources of information to aid their medical decision-making process were expected to be more likely to misjudge risks and overemphasise benefits of various treatments.

 Furthermore, all experts anticipated that these factors could influence asymmetry of risks and benefits bias and result in patients assuming that knowing more about their health status is better than knowing less (imperative knowledge bias).^76^ As a result, patients influenced by the “imperative knowledge bias” were anticipated to be more vulnerable to act (imperative action bias) and demand low-value care instead of doing nothing.

 Loop R6 “Practice-induced demand” consists of two factors that have a reinforcing positive effect on each other. This effect is interpreted either from bottom to top or from top to bottom. On the one hand, if more patients demand low-value care, this behaviour can become normalised as a societal custom or habit, resulting in increased demand for care.^69,70^ On the other hand, patients who regard demanding low-value care as a normal or a customary practice are more likely to demand low-value care. Furthermore, the experts concluded that patients’ expectations may be a crucial mechanism to explain this behaviour. Habitual practices can encourage expectations related to medical decision-making, and this can drive patients’ demand for low-value care.

 Loop R7 “Care entitlement” describes experts’ view of how “care entitlement” contributes to patients’ demand.^55,63^ Experiences shared by patients’ social network of receiving care when facing similar situations to that of the patients, can cause patients to feel entitled to receive the same treatment. In this manner, patients’ sense of entitlement may influence patients to receive care when this is in fact not needed or recommended.^77^

 Other reasons considered to cause this sense of entitlement were the factors “health insurance payments” and “not accepting the concept of overuse.” The former is related to the idea that health insurance is costly for patients and because of this, patients feel entitled to claim care.^21^ The latter concerns the notion of rejecting and/or questioning the credibility of evidence-based recommendations and care overuse messages used by healthcare providers to avoid low-value care.^17,77^ Both cases may result in patients feeling entitled to receive care, thinking that they are exceptions to the rule. For example, having different demographic characteristics in comparison to patients included in studies was mentioned as a common argument of patients to not accept clinical guidelines. Patients using this argument expected a higher chance of receiving benefits from care that was not recommended to them.

 Loop R8 “Social and media influence” includes a reinforcing positive effect between the experiences shared by social networks and patients’ sense of entitlement to receive care. This effect was argued to have a direct impact on demand for low-value care and could indirectly influence other factors in the CLD. Patients feeling entitled to demand care were expected to encourage or pressure their social network(s) to do the same.^55,63,70^ However, experiences shared within patients’ social networks may also contribute to patients’ sense of entitlement to receive and demand low-value care.

 Furthermore, the experts discussed how marketing strategies or media coverage may be an additional factor influencing patients’ entitlement sentiment. In these cases, information about low-value care interventions is presented or communicated in such a manner that it causes patients to seek this type of care.^10,49,78^ For example, magazine articles raising public awareness of available care options that should be used before a certain period passes can result in a sense of entitlement to receive care.

 Loop R9 “Patient-provider relationships” focuses on the relationship between patients’ “perceived insecurity” and “interaction with the provider.” Interpreting the loop from top to bottom, the factors concluded to influence patients’ insecurity are similar to the ones described in loop R1. However, our experts viewed the interaction with the provider as an additional cause and effect of patients’ “perceived insecurity” and therefore, created a causal loop.^79^ It was stated that an increased state of insecurity may interfere with interactions between patients and providers and cause patients to view this interaction as poor or negative. Negatively perceived interactions could be the result of patients not receiving their preferred care,^80^ resulting in them not feeling helped or supported and therefore becoming more insecure.^81^ Repetitions of this loop may continue to hinder interactions between the patient and provider and increase patients’ insecurity. Furthermore, the experts anticipated that a negative interaction with the provider may result in more demand for low-value care.

 The interaction with the provider was acknowledged as a crucial factor to regulate patients’ “perceived insecurity” and other factors in the CLD.^3,82^ Experts concluded that this factor is influenced by a chain of other factors that starts when providers experience uncertainty and communicate this to their patients.^82-84^ Providers can face all sorts of uncertainties, such as not being able to diagnose the patient, and therefore, not being able to help the patient. This “communication of uncertainty by providers” was argued to influence three mechanisms connected to patients’ “trust in the provider.”^85-87^ First, patients may view their provider as medically incompetent and lose trust in them (perception of providers’ medical competency). Second, patients could perceive their provider as honest and trust their provider more because of this (perception of providers’ integrity). Third, patients could assume that their provider is unable to act and help them further, causing them to lose trust in the provider (providers’ ability to act).^85-87^ This loss of trust was expected to decrease patients’ degree of authority acceptance, causing them to ignore recommendations of the healthcare professional more often.^87-89^ Thus, this further deteriorates the interaction with the provider.

 The “cultural background” of patients was anticipated as an additional factor influencing patients’ trust in the provider.^90,91^ In the context of low-value care, patients with various cultural backgrounds were expected to be less likely to accept recommendations made by their provider and refrain from use of low-value care. These patients may for instance visit physicians in their home country, where they hope to receive their preferred treatment. By receiving care from a different physician, patients may come to question the authority of physicians when they receive (low-value) care from physicians in their home country but not from care providers in other countries.^92^

 The remaining factors that were not directly connected to any feedback loop in the CLD were mostly associated with educational background.^93,94^ However, opposing mechanisms were discussed. On the one hand, patients with a higher educational level were believed to be more vocal and outspoken in demanding low-value care. These patients may be more able to clearly indicate to their provider what they want, resulting in a higher demand for low-value care. In contrast, patients with lower levels of health literacy were believed to also demand more care. Furthermore, patients with lower educational levels were also considered to have a generally higher demand for low-value care services. However, participants did not provide a mechanism supporting this conclusion.

## Discussion

 The aim of this study was to explore and map factors driving patients’ demand for low-value care and how they are related by using a complex systems approach. To this end, two GMB sessions were conducted with seven experts in total. The final CLD was constructed based on these GMB sessions and consisted of 42 factors contributing to patients’ demand for low-value care. We identified 59 relations between factors, which were either positive (ie, 51 of 59) or negative (ie, 8 of 59), as well as nine reinforcing feedback loops. Through our CLD, we provide a richer and more holistic illustration of how distinct factors interact with each other and affect patients’ demand for low-value care. This supports earlier work indicating that demand for low-value care can be perceived as a multifactorial or complex phenomenon.^2,10,22,23,26^

 The present work supports the notion that patients’ demand for low-value care is not a single actor problem, but a multi-agent or multilevel problem influenced by clinicians, patients, and healthcare systems.^95^ In this respect, the current CLD represents a complex subsystem existing next to subsystems that focus on factors driving the supply of low-value care by clinicians and/or creation of low-value care through system factors, which interact with each other. Factors at the edges of our CLD may be central factors in these other subsystems or “bridge factors” connecting to these subsystems.^96^ For example, the providers’ ability to act may be a central factor in the supply subsystem or a bridge factor connecting the demand and supply as low-value care subsystems. Gaining insight into the structure of these other subsystems related to low-value care, may provide further relevant insights for developing effective interventions to tackle low-value care.

 It is important to note some limitations of this study. First, we cannot claim that the final CLD presented in this paper contains a complete overview of all factors and relations that drive patients’ demand for low-value care. Because of the limited number of experts included, certain expertise or information may still be lacking from for example nurses, policy experts, and patient advocates. Therefore, there may be a certain degree of unexplored uncertainty of why patients demand low-value care in the final CLD. In this context, it is relevant to note that the exact structure of complex problems is difficult to determine and that modellers will always be confronted with uncertainties in the construction of a CLD.^40^ Therefore, a CLD should be seen as the visualisation of the joint mental models of participants in GMB sessions. More specifically, in this study, the final CLD should be regarded as a thorough exploration of patients’ demand for low-value care that researchers can use to formulate hypotheses, empirically assess the presumed relations, further validate the model, and search for potentially missed factors or relations.^24^

 The second limitation is related to the absence of balancing loops in the CLD. The participating experts suggested that these loops do not contribute to patients’ demand for low-value care and, therefore, balancing feedback loops may not have emerged in the GMB sessions. Another reason may be related to how the factors were named, as this can influence the polarity of connections and feedback loops.^40^ For example, labeling a factor as “trust in the provider” versus “distrust in the provider” may reverse the polarity of its relationships with other factors, thereby affecting the structure and interpretation of feedback loops.

 The presence of nine reinforcing feedback loops without any balancing feedback loops in the CLD, in theory, results in an ever-increasing demand for low-value care. This scenario may not be unlikely and is one of the reasons for this research. Nonetheless, there are other complex (sub)systems that may still discourage use of low-value care in practice. Where one system (our CLD) may drive demand upward, systems related to the supply or the healthcare system that focus on distribution, communication, and adherence to medical guidelines may drive use of low-value care downward. This emphasises the relevance of also studying these other subsystems, and the interactions between demand, supply, and the healthcare system.

 The third limitation concerns the applicability of the final CLD to specific low-value care cases. The aim of this study was to provide insight into the factors that influence patients’ demand for low-value care, and how they are related, in a generic sense. One could argue that a CLD constructed with this objective in mind is also applicable to disease or context-specific low-value care interventions that patients can demand. However, to what extent a generic CLD is applicable and sufficiently sensitive to specific low-value care demand cases remains unclear. Potentially, this may vary between contexts (eg, healthcare systems, disease areas, care settings), but also across different interventions within the same context. Determining the extent to which this is the case requires additional context-specific and comparative research.

 The fourth limitation involves the exclusion of patients during the GMB sessions. This was done to develop a CLD that provides general insight in patients’ demand for low-value care. While information from patients with specific diseases is useful, it may not be applicable to patients with other diseases. This could hinder the creation of a generic CLD concerning patients’ demand. Therefore, it was decided to exclude patients from this study. However, follow-up research could further refine the CLD by including perspectives of patients that have requested common low-value care practices. Examples include requests for antibiotics for viral infections, unnecessary imaging for non-specific low back pain or headaches, excessive laboratory testing for asymptomatic individuals, and demand for unnecessary screening or medication.^22^ For disease-specific CLDs, including patients in the study is highly recommended. For example, patients with low back pain should be included in a CLD study that explores factors and relations influencing patients demand for non-specific low back pain imaging, which is a form of patients’ demand for low-value care.^6^ Hereafter, it is recommendable to compare disease-specific CLDs to the generic CLD concerning patients’ demand to examine if these complex systems are similar.

 The last limitation concerns the difficulty to identify leverage points based on the structure of the CLD. These are points or variables in complex systems where small changes may results in significant changes in the whole CLD.^97^ From a complex systems perspective, Meadows^97^ argued that for policy interventions to be effective, they should focus on impacting leverage points in a complex system. The CLD contains several factors that have a high number of connections with other factors, which possibly makes them promising focal points for intervention within this complex system. Examples of such highly connected factors are perceived insecurity, asymmetry of risks and benefits bias, and entitlement to care (See Figure). Nonetheless, research concerning network analysis pointed out that focusing on the number of connections can lead to factors being wrongfully identified as leverage points for intervention in the system, particularly when this is not further validated by other methods.^98^ Although network analysis methods have not yet proven to be very effective at identifying leverage points, these methods do provide a first indication of factors that may be relevant. Factors identified through these methods may serve as starting points for research that aims to identify leverage points. Still, alternative methods to identify leverage points remain to be explored. We propose that further research should explore mixed method approaches for identifying leverage points. In addition to network analysis methods, sensitivity analyses could also provide more insight into the relative importance of distinct factors in a complex system and their connections, for example, by simulating the effect of changes in these factors on the variable of interest (here, patients’ demand for low-value care) using a computational system dynamics model.^40^

 In any case, this suggests that policy-makers should first grasp the factors and relations driving patients’ demand for low-value care before developing and implementing interventions. Based on the current CLD, a promising leverage point could be to reduce buffers and stocks. In this case this would refer to reducing available healthcare resources leading to this demand, such as the amount of practice variation.^97^ Decreasing buffers of available care services could discourage demand for low-value care. Another option is to add negative feedback loops to the system that can correct and stabilise reinforcing factors.^97^ Interventions that target feedback loops or factors within these loops may help disrupt or reduce their reinforcing effects on other factors and diminish demand for low-value care. Alternatively, interventions could focus on changing the underlying beliefs that drive system behaviour.^97^ For example, changing patients’ view that more care is always better may alter their care-seeking patterns. Another suggestion is to intervene at central drivers embedded in multiple feedback loops that underpin underlying beliefs in the CLD, such as perceived insecurity. This could be achieved through clinical guidelines and public education campaigns aimed at reducing uncertainty-driven demand.

 Such interventions in the status quo concerning demand for low-value care may lead to opposition among patients in the short term, but eventually people may be expected to grow accustomed to such changes. Over time, sustainable new habits or beliefs may be formed that could help reduce this demand.^97^ Patients may even become aware that their demand for low-value care is infuenced by other factors. That said, the question remains if and how these general recommendations to reduce patients’ demand will work in specific low-value care demand cases. Therefore, further analysis is needed to determine whether policies aimed at reducing low-value care in general will be equally effective (eg, more, or less) in specific cases.

 Concluding, this study provides an overview of factors that drive patients’ demand for low-value care and their interactions, based on GMB sessions with experts. The CLD offers insight into the factors, mechanisms, and feedback loops contributing to patients’ demand for low-value care. It can help to identify central drivers (eg, perceived insecurity) and feedback loops that influence many other factors, including patients’ demand for low-value care. The findings of this study may help policy-makers develop and implement more effective interventions to curb increasing demand for low-value care. For example, interventions could focus on the central drivers, feedback loops, or underlying beliefs in the CLD. Further research is recommended to validate the factors and connections in the CLD, as well as to examine the supply and healthcare system factors affecting low-value care demand and the interactions between these systems.

## Disclosure of artificial intelligence (AI) use

 We used the AI tool ChatRIVM (an in-company version of ChatGPT) to translate the relevant Dutch text fragments from the transcripts in Supplementary file 2 to English.

## Ethical issues

 The Centre for Clinical Expertise (CCE) at the National Institute for Public Health and the Environment (RIVM) in the Netherlands approved the study (reference G&Z-723) according to the National (Dutch) and European legislation. The study was conducted in accordance with the principles of the Declaration of Helsinki, the Dutch Code of Conduct for Health Research, and Regulation (EU) 2016/679 (General Data Protection Regulation). The study focused on gathering expert perspectives and knowledge regarding the factors and their relations influencing patients’ demand for low-value care to develop a conceptual model. For this reason, we did not collect personal, identifiable, or sensitive data from human participants. Additionally no interventions were conducted that could pose harm or risk to individuals. Informed consent was obtained from all participants before collecting data.

## Conflicts of interest

 Authors declare that they have no conflicts of interest.

## Supplementary files


Supplementary file 1. Topic List Based on Scoping Review.

Supplementary file 2. Words and Arrow Diagram.

Supplementary file 3. Definitions of Factors in This Study.
