# Supplementary material for: Exploring and Mapping Expert Views on the Mechanisms Contributing to Patients’ Demand for Low-Value Care: A Complex Systems Approach
Source: Int J Health Policy Manag. 2026 Jun 1;15:9099. doi: 10.34172/ijhpm.9099 (PMC13729795; doi:10.34172/ijhpm.9099)
Supplement: Supplementary file 1 [file ijhpm-15-9099-s001.pdf]

Article title: Exploring and Mapping Expert Views on the Mechanisms Contributing to Patients' Demand for Low-Value Care: A Complex Systems Approach

Journal name: International Journal of Health Policy and Management (IJHPM)

Authors' information: Gillroy R. L. Fraser[1,2,3,4]*, G. Ardine de Wit[1,2], Job van Exel[3,4], Peter M. A. Sloot[5,6], Raymond W. J. G. Ostelo[2,7], Frenk van Harreveld[8], Mattijs S. Lambooij[3,4,9]

[1]Department of Health Economics and Health Services Research, National Institute for Public Health and the Environment (RIVM), Bilthoven, The Netherlands.

[2]Department of Health Sciences, Faculty of Science, Vrije Universiteit Amsterdam, Amsterdam Public Health Research Institute, Amsterdam, The Netherlands.

[3]Erasmus School of Health Policy & Management, Erasmus University Rotterdam, Rotterdam, The Netherlands.

[4]Erasmus Centre for Health Economics Rotterdam (EsCHER), Rotterdam, The Netherlands.

[5]Institute for Advanced Study, University of Amsterdam, Amsterdam, The Netherlands.

[6]Independent Complex Systems Researcher, Noordwijk, The Netherlands.

[7]Department of Epidemiology and Data Science, Amsterdam University Medical Centre, Vrije Universiteit, Amsterdam, The Netherlands.

[8]Department of Psychology, Faculty of Social and Behavioral Sciences, University of Amsterdam, Amsterdam, The Netherlands.

[9]Center for Prevention, Lifestyle and Health, Department Behaviour & Health, National Institute for Public Health and the Environment (RIVM), Bilthoven, The Netherlands.

***Correspondence to:** Gillroy R. L. Fraser; Email: gillroy.fraser@rivm.nl

Citation: Fraser GRL, de Wit GA, van Exel J, et al. Exploring and mapping expert views on the mechanisms contributing to patients' demand for low-value care: a complex systems approach. Int J Health Policy Manag. 2026;15:9099. doi:10.34172/ijhpm.9099

Supplementary file 1. Topic List Based on Scoping Review

(Fraser et al., 2024 Under review)

**Factor**	**Definition**
Cognitive biases	A systematically occurring tendency to think, act or feel in a certain manner, caused by processing and interpreting information derived from individual experiences and preferences.
Anchoring effect	Anchoring effect occurs when patients rely on initially found or received information, instead of relying on evidence that is of a higher quality but also more difficult to find.
Anticipated regret aversion bias	Anticipated regret aversion bias includes instances where patients strive to avoid possible regret in the future by demanding unnecessary care in the present.

Asymmetry of risks and benefits bias	Asymmetry of risks and benefits bias is a tendency of patients to overestimate the benefits and underestimate the risks of low-value care treatments.
Confirmation bias	The confirmation bias occurs when patients seek or interpret medical information or care recommendations that correspond to their viewpoints, and disregard or dispute contradicting recommendations and information. Furthermore, patients influenced by this bias can prefer more healthcare than needed, and ignore, reject, or give less attention to information about the harms of screening and overdiagnosis.
Extension bias	The extension bias is the tendency of patients to perceive more healthcare as better than less healthcare. Patients incentivized by this bias can demand excessive, unnecessary, and even harmful care.
Imperative action bias	Patients with this bias feel obliged to act because action is better than inaction.
Imperative knowledge bias	A tendency of patients to gain more information related to their health status, because this is better than being ignorant. Therefore, patients tend to think that to know is better than not to know. This bias occurs primarily in the field of diagnostics, and usually leads to overdiagnosis.
Loss aversion	Loss aversion occurs when patients overvalue low-value care options and experience discomfort with losing a preferred care option. These patients tend to continue demanding low-value care even when physicians do not recommend this, because the losses loom larger than the gains.
Prominence effect	Patients can perceive medical decision-making as complex and challenging because of numerous factors that must be considered. Because of this, patients may try to resolve this complexity by focusing on a single aspect that for some reason is salient to them.
Risk aversion	A tendency of patients to avoid or reduce uncertainties, dangerous situations, and risks as much as possible
**Emotions**	Research in the domain of psychology has long established that many types of (health-related) behaviors are to a significant extent driven by emotions.
Fear and anxiety	Being afraid of illness, overlooking potential diseases, or possible outcomes of diseases. To reduce these fears and anxieties, patients are more likely to demand low-value care.
Need for control	Patients strive to assert control over their situation by displaying an active role in their decision-making process. For example, patients might try to gather as much information as they can regarding their situation and act upon it by demanding care, even when this is unnecessary.
Perceived insecurity	Perceived insecurity causes patients to feel uncertain about their health status. Patients then may wish to reassure themselves by demanding care, including low-value care.
**Preferences and expectations**	Personal convictions and past experiences of patients associated with the demand low-value care.

Beliefs	Patients' beliefs are formed by personal convictions, which function as cornerstones that shape patients' attitudes, preferences, and expectations towards medical care. These beliefs tend to make patients susceptible to perceive low-value care as a default or necessary treatment, which causes these patients to demand low-value care.
Experiences	Past events that shape or influence their preferences and expectations to demand medical care.
**Knowledge-related factors**	All information and education-related factors that drive patients to demand low-value care.
Cognitive limitation	When patients lack sufficient ability to comprehend information about low-value care treatments and services. For example, patients lacking sufficient knowledge, tertiary education, or (medical) health literacy can demand low-value care However, there are also examples of patients with higher education levels demanding low-value care.
Over-informed	Instances where patients possess too much information, resulting in a sense of data overload and wrongful healthcare decision-making.
Unawareness	In some cases, patients lack realization and information that certain treatments have no or only marginal health benefits and are possibly even harmful. Because of their 'unawareness' patients are often surprised to learn about the downsides of interventions characterized as low-value care.
Not accepting the concept of overuse	Tendencies of patients to reject or question the credibility of evidence-based recommendations and overuse messaging to avoid low-value care interventions, such as overtreatment and overdiagnosis
**Interaction with the healthcare provider**	All factors related to the interaction with healthcare providers that result in the demand for low-value care
Acceptance of care recommended by the provider	Instances where patients adhered to low-value care recommendations provided by their healthcare providers
Lack of trust in the provider	It is hypothesized that patients sometimes have less trust in the diagnostic capabilities of the primary care physician than of medical specialists and, therefore, ask to be referred to a medical specialist.
**Socio-cultural factors**	All factors related to social or cultural incentives for patients to demand low-value care.
Entitlement to care	The idea that patients perceive healthcare as a right. For instance, patients justify low-value care demand when healthcare is experienced as costly. Because of this, patients have higher expectations of care and are more willing to consume unnecessary and excessive healthcare. In other cases, the existence of public standards or social norms determine this sense of care entitlement.
Social network	Instances where relatives and acquaintances pressure or encourage patients to seek low-value care.

Stage of life	Age- and life phase related factors that result in patients' low-value care demand. For example, some individuals perceive that being young of age justifies more aggressive unnecessary treatment.
**Biomedical and care-related factors**	Factors on the intersection of biology, care services and medicine.
Duration of symptom or illness	The idea that a persisting or reoccurring medical problem, symptom, or disease, contributes to excessive demand for low-value care.
Maximization of length and quality of life	The wish of patients to live as long as possible at any cost, and to maintain or improve their current quality of life.
Pain	Pain experienced by patients was observed to be a factor for the prescription of inappropriate medication and unnecessary treatment by the provider.
Severity and number of health threats	Patients experiencing multimorbidity, severe symptoms or illnesses have an increased tendency to demand low-value care.
Ease of use	The idea of demanding care because it is convenient (e.g., nearby), time efficient, or perceiving other options as uncomfortable by the patient. These reasons may contribute to the appeal and demand of various types of low-value care by patients.
**Economic factors**	Factors relating to the consumption of low-value care for financial reasons or to satisfy needs and wants of patients.
Consumerism	The idea that healthcare is perceived as a consumption good, and patients can, therefore, shop around by visiting various healthcare providers to receive their desired care.
Present and future income effects	The need of patients to function at work to earn money and support their family. For this reason, patients are more likely to request low-value care when they are injured or sick
Marketing	When information is presented in such a manner that it generates profits for the presenting actor and causes patients to demand low-value care. In some cases, campaigns or advertisements expose consumers solely to the potential benefits of low-value care services. In other cases, patients are misled by awareness campaigns about certain medical conditions overemphasizing the number of patients suffering from a certain disease. Consequently, in the eyes of the public, some diseases are perceived as more common or more serious than they actually are.
Insurance coverage	Demanding low-value care because it is covered by patients' insurance and, therefore, patients do not directly bear the financial consequences of care use.
