# Supplementary material for: Exploring and Mapping Expert Views on the Mechanisms Contributing to Patients’ Demand for Low-Value Care: A Complex Systems Approach
Source: Int J Health Policy Manag. 2026 Jun 1;15:9099. doi: 10.34172/ijhpm.9099 (PMC13729795; doi:10.34172/ijhpm.9099)
Supplement: Supplementary file 2 [file ijhpm-15-9099-s003.pdf]

Article title: Exploring and Mapping Expert Views on the Mechanisms Contributing to Patients' Demand for Low-Value Care: A Complex Systems Approach

Journal name: International Journal of Health Policy and Management (IJHPM)

Authors' information: Gillroy R. L. Fraser[1,2,3,4]*, G. Ardine de Wit[1,2], Job van Exel[3,4], Peter M. A. Sloot[5,6], Raymond W. J. G. Ostelo[2,7], Frenk van Harreveld[8], Mattijs S. Lambooij[3,4,9]

[1]Department of Health Economics and Health Services Research, National Institute for Public Health and the Environment (RIVM), Bilthoven, The Netherlands.

[2]Department of Health Sciences, Faculty of Science, Vrije Universiteit Amsterdam, Amsterdam Public Health Research Institute, Amsterdam, The Netherlands.

[3]Erasmus School of Health Policy & Management, Erasmus University Rotterdam, Rotterdam, The Netherlands.

[4]Erasmus Centre for Health Economics Rotterdam (EsCHER), Rotterdam, The Netherlands.

[5]Institute for Advanced Study, University of Amsterdam, Amsterdam, The Netherlands.

[6]Independent Complex Systems Researcher, Noordwijk, The Netherlands.

[7]Department of Epidemiology and Data Science, Amsterdam University Medical Centre, Vrije Universiteit, Amsterdam, The Netherlands.

[8]Department of Psychology, Faculty of Social and Behavioral Sciences, University of Amsterdam, Amsterdam, The Netherlands.

[9]Center for Prevention, Lifestyle and Health, Department Behaviour & Health, National Institute for Public Health and the Environment (RIVM), Bilthoven, The Netherlands.

***Correspondence to:** Gillroy R. L. Fraser; Email: gillroy.fraser@rivm.nl

Citation: Fraser GRL, de Wit GA, van Exel J, et al. Exploring and mapping expert views on the mechanisms contributing to patients' demand for low-value care: a complex systems approach. Int J Health Policy Manag. 2026;15:9099. doi:10.34172/ijhpm.9099

Supplementary file 3. Definitions of Factors in This Study

Core theme	Factors	Definition
Biomedical and care-related factors	Chronic problem	A persisting or reoccurring medical problem, symptom, or disease.
Biomedical and care-related factors	Salient health problem	A noticeable or relevant health issue, symptom, or disease.

Core theme	Factors	Definition
Biomedical and care related factors	Severity of health problem	The intensity of the experienced health issue.
Cognitive biases	Anticipated regret aversion bias	A tendency to avoid possible regret in the future by demanding unnecessary care in the present.
Cognitive biases	Asymmetry of risks and benefits bias	A tendency to overestimate the benefits and underestimate the risks of low-value care treatments.
Cognitive biases	Confirmation bias	A tendency to seek or interpret medical information or care recommendations that correspond to their viewpoints, and disregard or dispute contradicting recommendations and information.
Cognitive biases	Imperative action bias	A tendency to feel obliged to act because action is better than inaction.
Cognitive biases	Imperative knowledge bias	A tendency to gain more information related to their health status, because this is better than being ignorant.
Cognitive biases	Publication bias	A tendency of researchers or publishers to focus on the positive research results and to neglect or negative or non-significant results.
Cognitive biases	Risk aversion	A tendency of patients to avoid or reduce uncertainties, dangerous situations, and risks as much as possible.
Economic factors	Consumerism	The idea that healthcare is perceived as a consumption good, and patients can, therefore, shop around by visiting various healthcare providers to receive their desired care.
Economic factors	Health insurance payments	Contributions that are paid to be insured for medical expenses.
Economic factors	Income abundance	Having a substantial amount of wealth or financial prosperity that can be spent on unnecessary treatments. For example, spending money on an unnecessary full body scan.
Economic factors	Marketing and media	Presenting information in such a manner that it generates profits for the presenting actor and causes patients to demand low-value care.
Emotions	Fear and anxiety	When patients are afraid of uncertainties, such as illnesses, overlooking potential diseases, or possible outcomes of diseases.
Emotions	Loss of control	To assert control over their situation by displaying an active role in their decision-making process.
Emotions	Perceived insecurity	Patients feel uncertain about their health status.

Core theme	Factors	Definition
Interaction with the provider	Providers’ ability to act	The possibility of the healthcare provider to provide care.
Interaction with the provider	Authority acceptance	Acknowledging and embracing recommendations and care provided by the healthcare provider
Interaction with the provider	Communication of uncertainty by providers	When healthcare providers discuss uncertainties with their patients related to the patient's experienced health problem or symptoms.
Interaction with the provider	Interaction with the provider	The process where patients and providers communicate and interact with each other.
Preferences and expectations	Patients’ expectations	When patients think or expect that their healthcare provider wants to provide care.
Interaction with the provider	Perception of providers’ integrity	Perception of patients about the integrity of healthcare providers.
Interaction with the provider	Perception of providers’ medical competency	Perception of patients about the capacities of healthcare providers.
System factor	Practice variation	Differences in recommendations of care by various healthcare providers
Preferences and expectations	Provider expectations	When healthcare providers think or expect that their patients want to receive care.
Interaction with the provider	Trust in the provider	The reliance and belief that patients have in their healthcare provider.
Knowledge-related factors	Demand of low-educated patients	When low-educated patients demand low-value care.
Knowledge-related factors	Health literacy	The ability of patients to comprehend medical or health-related information.
Knowledge-related factors	High education	Patients with a degree that is valued as above average.
Knowledge-related factors	Lack of evidence	When information about the harmful or marginal effect of low-value care is absent or insufficient.
Knowledge-related factors	Not accepting the concept of overuse	Rejecting or questioning the credibility of evidence-based recommendations and overuse messaging to avoid low-value care interventions, such as overtreatment and overdiagnosis
Knowledge-related factors	Patients’ outspokenness	The assertiveness and vocalness of patients to demand low-value care.
Knowledge-related factors	Quality of distributed information to patients	The quality of Information about treatments and services that is provided to patients.

Core theme	Factors	Definition
Knowledge-related factors	Unawareness	Lacking realization and information that certain treatments have no or only marginal health benefits and are possibly even harmful.
Preferences and expectations	Beliefs	Patients' beliefs are formed by personal convictions, which function as cornerstones that shape patients' attitudes, preferences, and expectations towards medical care. These beliefs tend to make patients susceptible to perceive low-value care as a default or necessary treatment, which causes these patients to demand low-value care
Socio-cultural factors	Experiences of social network	When relatives and acquaintances pressure or encourage patients to seek low-value care by sharing their personal observations or experiences.
Socio-cultural factors	Cultural background	The influence of foreign medical practices that influence patients to demand low-value care in the Netherlands
Socio-cultural factors	Entitlement to care	When patients consume unnecessary and excessive healthcare because this is perceived as a right.
Socio-cultural factors	Habitual practices	Medical-related customs or habits that are common for patients to receive care
Socio-cultural factors	Stage of life	Age- and life phase related factors that result in patients' low-value care demand.
Dependent variable	Demand for low-value care	When patients demand care that is potentially harmful and provides little to no health benefits to patients.
